# Supplementary material for: Counting cells can accurately predict small-molecule bioactivity benchmarks
Source: Nat Commun. 2026 Feb 6;17:2436. doi: 10.1038/s41467-026-68725-5 (PMC12988037; doi:10.1038/s41467-026-68725-5)
Supplement: Supplementary file 1 — Supplementary Information [file 41467_2026_68725_MOESM1_ESM.pdf]

Supplementary Information

Counting Cells Can Accurately Predict Small-Molecule Bioactivity Benchmarks

Srijit Seal[1,2+]*, William Dee[3+], Adit Shah[2], Natacha Cerisier[4], Andrew Zhang[5], Esteban Miglietta[2], Katherine Titterton[6], Ángel Alexander Cabrera[6], Daniil Boiko[6], Alex Beatson[6], Gregory Slabaugh[3], Olivier Taboureau[4], Jordi Carreras Puigvert[7,8], Shantanu Singh[2], Ola Spjuth[7,8]*, Andreas Bender[9,10,1]*, Anne E. Carpenter[2]*

1 Department of Chemistry, University of Cambridge, Cambridge, UK

2 Broad Institute of MIT and Harvard, Cambridge, MA, US

3 Digital Environment Research Institute (DERI), Queen Mary University of London, London, UK

4 Université Paris Cité, INSERM U1133, CNRS UMR 8251, 75013, Paris, France

5 Health Sciences and Technology, Harvard-MIT, Cambridge, MA, US

6 Axiom Bio, San Francisco, CA, US

7 Department of Pharmaceutical Biosciences and Science for Life Laboratory, Uppsala University, Uppsala, Sweden

8 Pixl Bio AB, Uppsala, Sweden

9 College of Medicine and Health Sciences, Khalifa University of Science and Technology, Abu Dhabi, United Arab Emirates

10 STAR-UBB Institute, Babeş-Bolyai University, Cluj-Napoca, Romania

[+] These authors contributed equally

Corresponding Author: srijit@understanding.bio, andreas.bender@ku.ac.ae, ola.spjuth@uu.se, anne@broadinstitute.org

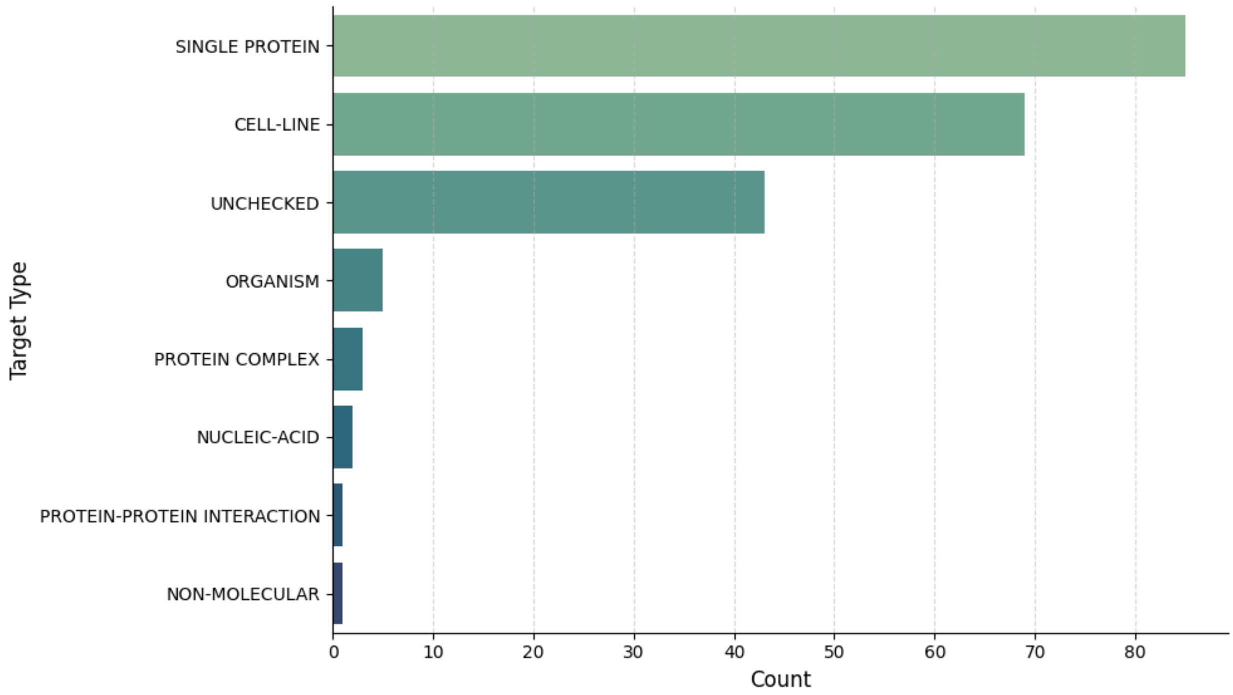

Figure S1. Distribution of target types across all assays in the Hofmarcher dataset. Each bar represents the number of assays associated with a specific target type, such as single proteins, cell-line (viability) assays.

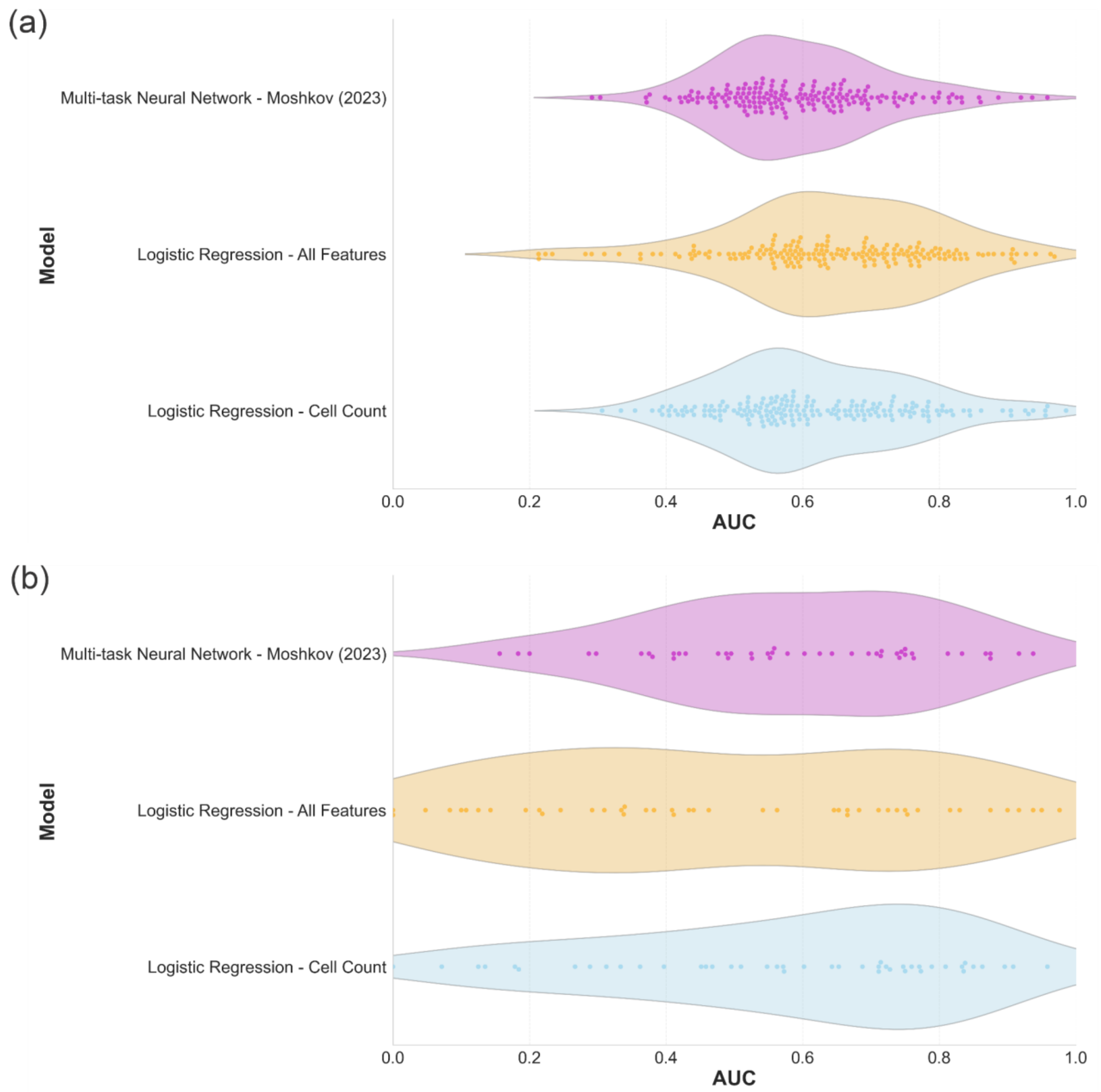

Figure S2. Distribution of performance of three different models across the assays (dots) when; (a) there are >= five active compounds, (b) < five active compounds in the tested assay. The logistic regression models perform comparably to the more sophisticated multi-task neural network in both scenarios, but (b) demonstrates that, in the case of assays with limited active compounds, using cell count only can lead to superior prediction results.

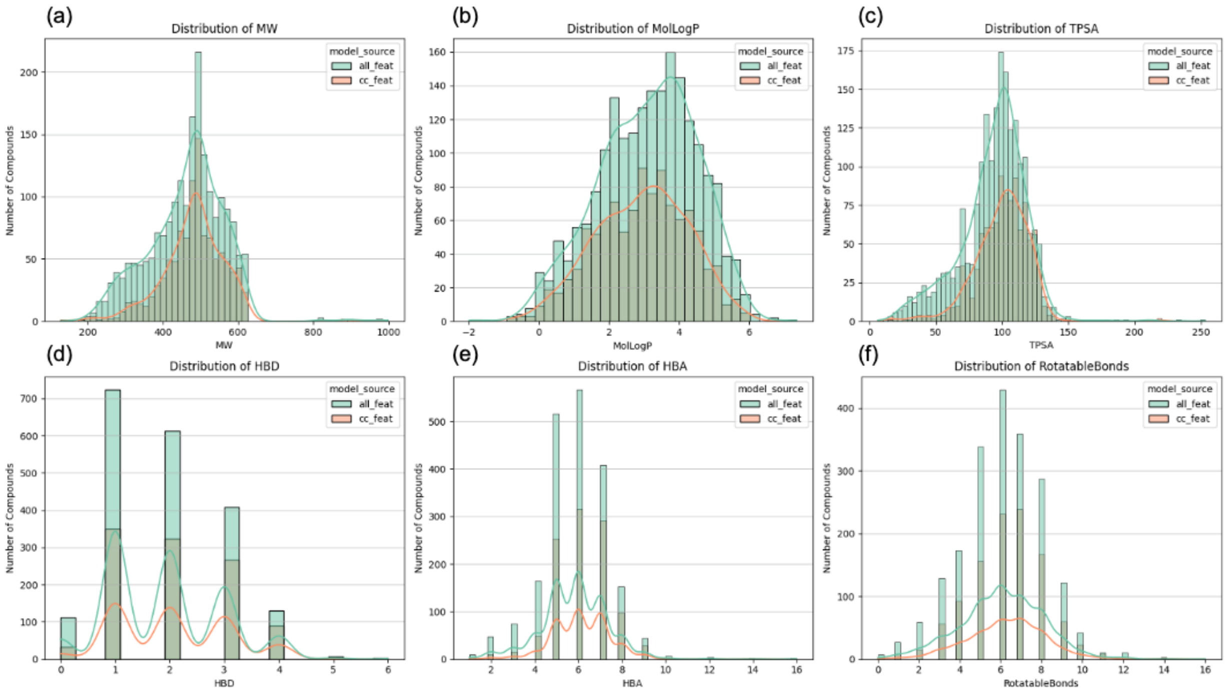

Figure S3. Distribution of physicochemical properties for uniquely predicted true positive compounds from each model. Histograms compare the distributions of (A) molecular weight (MW), (B) lipophilicity (LogP), (C) topological polar surface area (TPSA), (D) number of hydrogen bond donors (HBD), (E) number of hydrogen bond acceptors (HBA), and (F) number of rotatable bonds between compounds uniquely predicted as true positives by the Cell Count Feature Model (cc_feat) and the All Feature Model (all_feat). Statistical comparisons were conducted using independent t-test; features with $p < 0.05$ are considered significantly different.

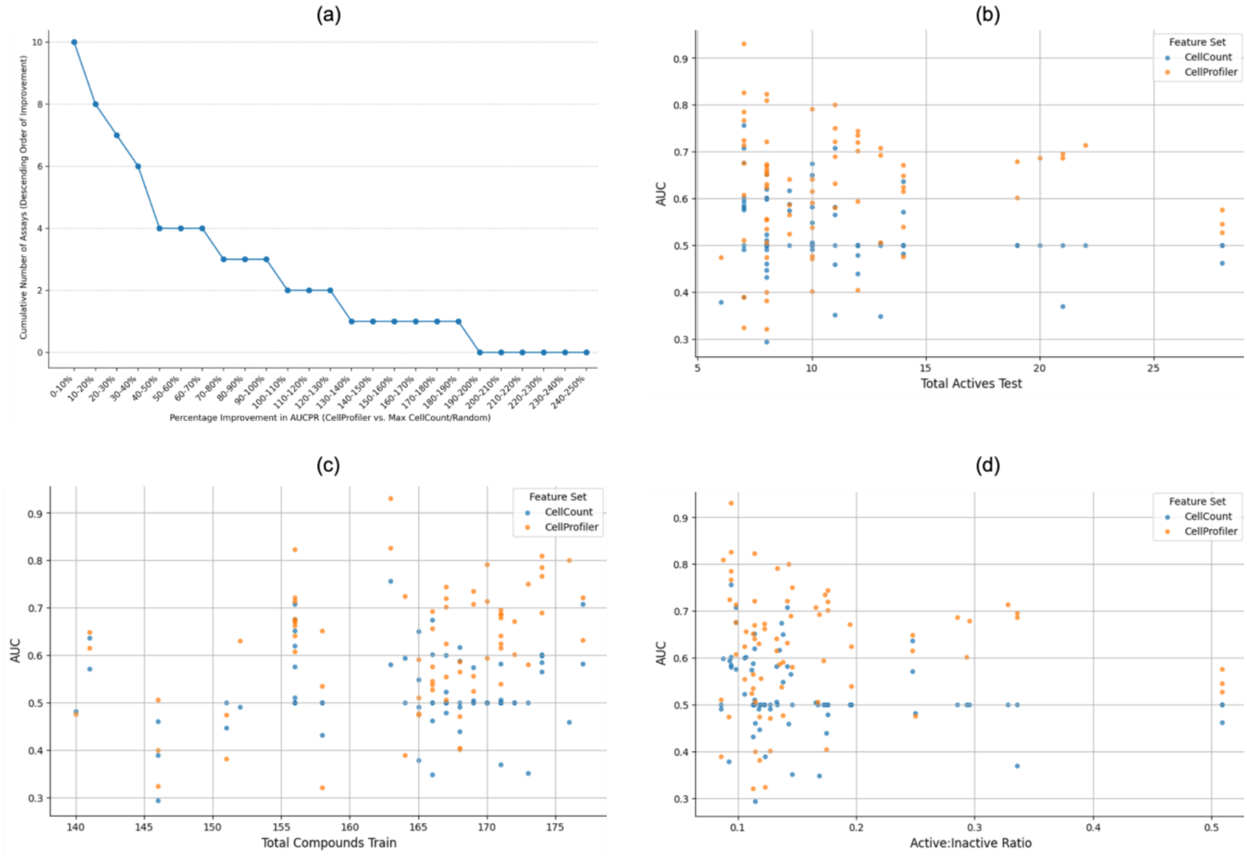

Figure S4. (a) Number of Assays out of 24 studied in the EveBio dataset where we observed a percentage improvement in AUCPR when using CellProfiler features compared to the best of a baseline cell count model of random shuffling or a baseline cell count model using only the cell count feature. Distribution of AUCROC achieved by model comapred to (b) total active compouines in the test dataset, (c) total compounds in the training data, and (d) the ratio of active to inactive compounds in the dataset. All dataset characteristics are mean of three folds, where given stratified splits result in individual characteristics of folds to be the same.

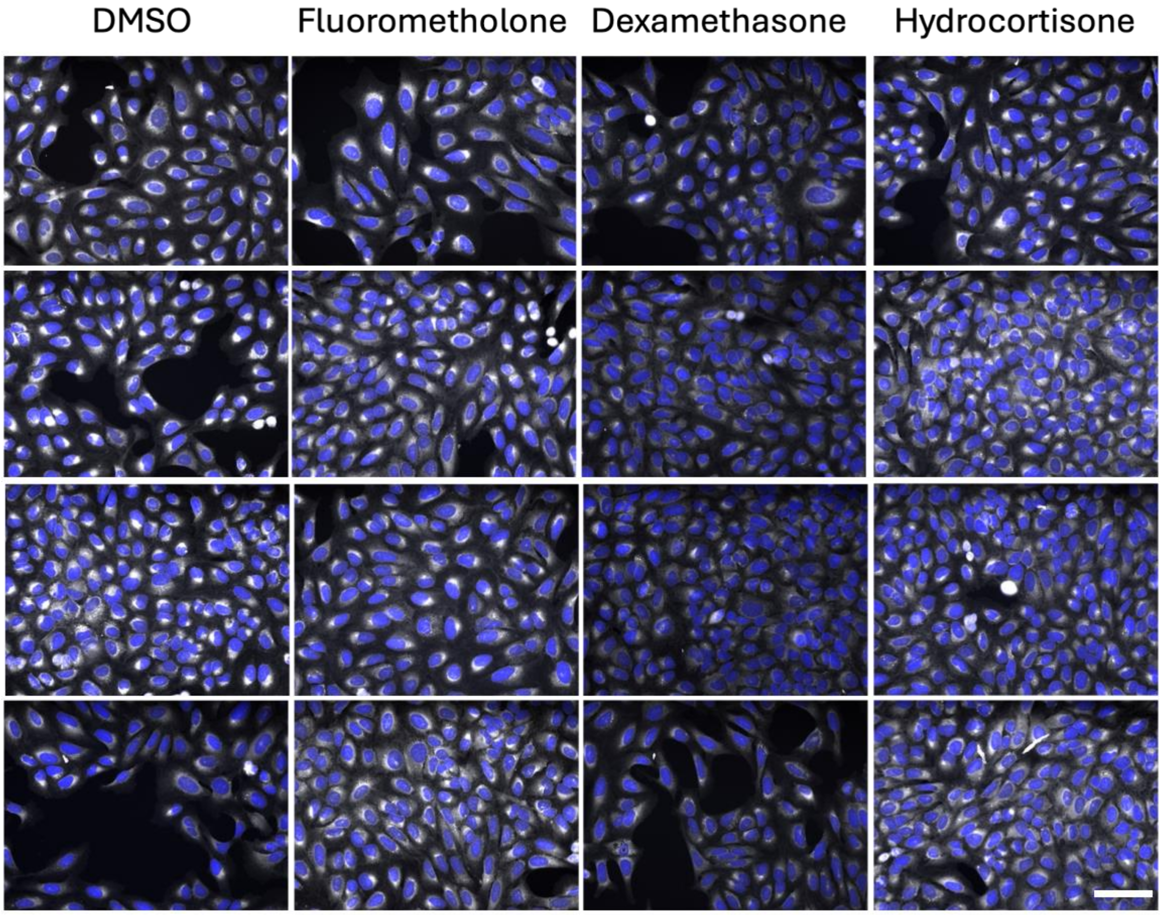

Figure S5. Representative Cell Painting images of GR agonist and DMSO-treated cells stained for ER (white) and nuclei (DAPI, blue). DMSO (vehicle control), Fluorometholone, Dexamethasone, and Hydrocortisone are shown with multiple replicate images (rows). Scale bar: 100 μm.

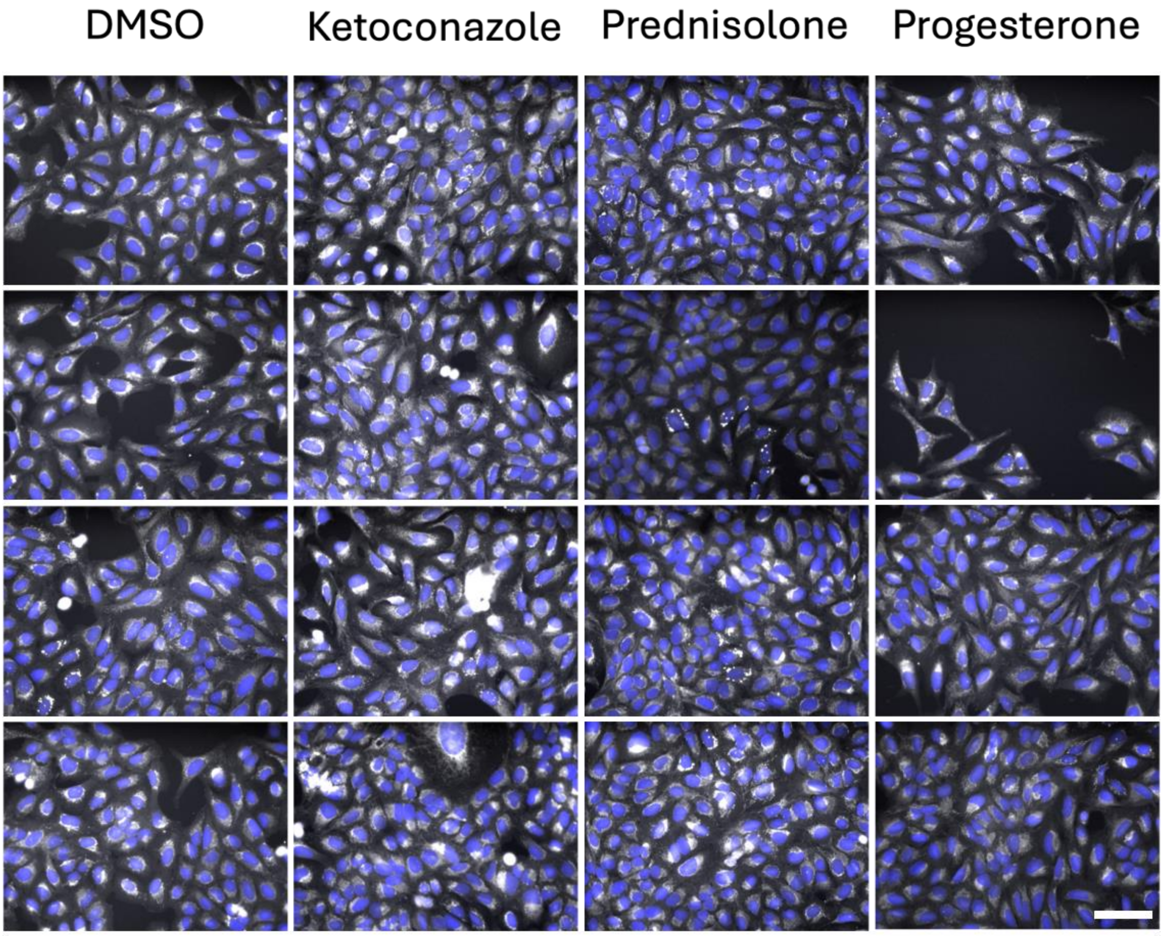

Figure S6. Representative Cell Painting images of RARb antagonist and DMSO-treated cells stained for mitochondria (white) and nuclei (DAPI, blue). DMSO (vehicle control), Ketoconazole, Prednisolone, and Progesterone perturbations are shown with multiple replicate images (rows). Scale bar: 100 µm.
