# Supplementary material for: Counting cells can accurately predict small-molecule bioactivity benchmarks
Source: Nat Commun. 2026 Feb 6;17:2436. doi: 10.1038/s41467-026-68725-5 (PMC12988037; doi:10.1038/s41467-026-68725-5)
Supplement: Supplementary file 2 — Description of Additional Supplementary Files [file 41467_2026_68725_MOESM2_ESM.docx]

**Description of Supplementary Data.**

Supplementary Data 1. Annotated assays from the Hofmarcher dataset with associated metadata including assay type, target type, organism, and a assay category.

Supplementary Data 2. Comparison of Protonet CP+ model (at support set size 64) with baseline cell count model as benchmarked on FSL-CP in Ha et al.

Supplementary Data 3. GO terms and description, Gene Ontology enrichment analysis of the selected compounds.
